# Genomic characteristics of invasive mucinous adenocarcinoma of the lung with multiple pulmonary sites of involvement

**DOI:** 10.1038/s41379-021-00872-0

**Published:** 2021-07-21

**Authors:** Moonsik Kim, Jinha Hwang, Kyung A Kim, Sohyun Hwang, Hye-Jeong Lee, Ji Ye Jung, Jin Gu Lee, Yoon Jin Cha, Hyo Sup Shim

**Affiliations:** 1 Department of Pathology, Kyungpook National University Chilgok Hospital, Kyungpook National University School of Medicine, Daegu, Republic of Korea; 2 Macrogen Inc., Seoul, Republic of Korea; 3 Department of Laboratory Medicine, Korea University Anam Hospital, Seoul, Republic of Korea; 4 Department of Pathology, Yonsei University College of Medicine, Seoul, Republic of Korea; 5 Department of Pathology, CHA University School of Medicine, Seongnam, Republic of Korea; 6 Department of Radiology and Research Institute of Radiological Science, Severance Hospital, Yonsei University College of Medicine, Seoul, Republic of Korea; 7 Division of Pulmonology, Department of Internal Medicine, Severance Hospital, Yonsei University College of Medicine, Seoul, Republic of Korea; 8 Department of Thoracic and Cardiovascular Surgery, Severance Hospital, Yonsei University College of Medicine, Seoul, Republic of Korea

**Keywords:** Non-small-cell lung cancer, Cancer genomics

## Abstract

Invasive mucinous adenocarcinoma (IMA) of the lung frequently presents with diffuse pneumonic-type features or multifocal lesions, which are regarded as a pattern of intrapulmonary metastases. However, the genomics of multifocal IMAs have not been well studied. We performed whole exome sequencing on samples taken from 2 to 5 regions in seven patients with synchronous multifocal IMAs of the lung (24 regions total). Early initiating driver events, such as *KRAS, NKX2-1, TP53, or ARID1A* mutations, are clonal mutations and were present in all multifocal IMAs in each patient. The tumor mutational burden of multifocal IMAs was low (mean: 1.13/mega base), but further analyses suggested intra-tumor heterogeneity. The mutational signature analysis found that IMAs were predominantly associated with endogenous mutational process (signature 1), APOBEC activity (signatures 2 and 13), and defective DNA mismatch repair (signature 6), but not related to smoking signature. IMAs synchronously located in the bilateral lower lobes of two patients with background usual interstitial pneumonia had different mutation types, suggesting that they were double primaries. In conclusion, genomic evidence found in this study indicated the clonal intrapulmonary spread of diffuse pneumonic-type or multifocal IMAs, although they can occur in multicentric origins in the background of usual interstitial pneumonia. IMAs exhibited a heterogeneous genomic landscape despite the low somatic mutation burden. Further studies are warranted to determine the clinical significance of the genomic characteristics of IMAs in expanded cohorts.

## Introduction

Lung cancer is the leading cause of cancer-related deaths worldwide. The most common subtype of lung cancer is adenocarcinoma [1]. Invasive mucinous adenocarcinoma (IMA) accounts for approximately 5% of all lung adenocarcinoma cases [2]. IMA is well known for its unique clinicopathologic characteristics [3]. Patients with IMA usually present with diffuse pneumonic patterns that manifest as multiple lung lesions [4]. Although recurrence in the lungs is frequently observed, extrathoracic or nodal metastasis can occur [5]. Histologically, IMA is characterized by goblet or columnar cells containing intracellular mucin with basally located nuclei [3]. Immunohistochemistry showed the loss of TTF-1/NKX2-1 protein expression, the lineage-specific protein, which is frequently used to distinguish between lung and other organ adenocarcinomas, in more than 80% of IMAs [6, 7]. *KRAS* mutations are the most common type of oncogenic driver mutations [5, 7–9]. *KRAS* G12D mutations are the most common subtype of *KRAS* mutations, followed by G12V, G12C, and G12A mutations [5, 9]. In addition to *KRAS* mutations, *BRAF* and *ERBB2* mutations, and gene rearrangements of *NRG1* have also been observed [5, 9–12].

Lung cancers with multiple lesions are challenging to classify. Generally, lung cancers involving multiple pulmonary sites occur in one of four patterns: (1) second primary cancers, (2) separate tumor nodules, (3) multifocal ground glass or lepidic nodules, and (4) diffuse pneumonic-type [4]. IMAs are usually classified as diffuse pneumonic-type cancers. In CT scans, they usually exhibit a diffuse consolidative pattern in the absence of proximal bronchial obstructions [4, 13, 14]. Histologically, IMAs are regarded as occurring through intrapulmonary metastasis and have a poorer prognosis than ground glass or lepidic nodules [4]. However, the genomic basis of this classification has not been well studied. While this study is in progress, only one study on comparative molecular profiling of spatially separate IMAs has been recently reported by Yang and colleagues [15]. The genomic structures of IMAs with multiple pulmonary sites of involvement are important because such knowledge can increase the accuracy of stage classifications, prognoses, and treatment plans. In this study, the genomics of spatially separated regions of multifocal IMAs were examined using whole exome sequencing (WES).

## Methods and materials

### Patients

Patients with IMAs who underwent surgical resection were enrolled from January 2014 to December 2018 at our institution. The institutional review board approved this retrospective study (No. 4-2019-0468). Clinical data including age, gender, smoking history, and radiologic findings were retrieved from electronic medical records.

### Histological review

Surgical specimens were fixed in 10% neutral-buffered formalin and embedded in paraffin blocks. For each formalin-fixed, paraffin-embedded (FFPE) tissue block, 4 μm sections were cut and stained with hematoxylin and eosin. IMA was diagnosed based on the 2015 World Health Organization classification [2].

### Whole exome sequencing

DNA was extracted from FFPE tissue using the QIAamp DNA FFPE Tissue kit according to the manufacturer’s protocols (Qiagen, Valencia, CA, USA). The concentration and quality of DNA were assessed using a NanoDrop spectrophotometer (Thermo Fisher Scientific, Waltham, MA, USA). WES was performed using the SureSelect Human All Exon V6 kit (Agilent Technologies, Santa Clara, CA, USA) and processed on the HiSeq 2500 platform to obtain a mean depth of 200× (Illumina, San Diego, CA, USA). Sequence reads were aligned to the human reference genome hg19 using the Burrows−Wheeler Aligner-MEM algorithm [16]. Duplicate or low-quality reads were marked using sambamba [17] and base quality score recalibration was performed using a genome analysis tool kit (GATK) [18].

### Somatic mutation calling

Somatic mutations were called using GATK MuTect2 using its default settings by comparing the sequences of tumor samples with those of matched normal samples [19]. Variant filtration functions in the GATK, such as FilterMutectCalls, CollectSequencingArtifactMetrics, and FilterByOrientationBias, were applied for the confident somatic calls. Somatic variants were annotated using the SnpEff and SnpSift functions [20]. To reduce the effect of false-positive variants, the following variants were excluded: (i) variants with a minor allele frequency of more than 1% in the genome aggregation [21], Exome Aggregation Consortium [22], and Korean population databases, (ii) variants with oxidized guanine to 8-oxoguanin (OxoG) artifacts, (iii) variants with mutated read counts less than 3, (iv) variants with total read depth less than 20, and (v) variants with a variant allele frequency (VAF) less than 3%. A manual review of *KRAS* codons 12, 13, and 61 for mutations below the VAF threshold for calling was performed using Integrated Genomics Viewer.

### Phylogenetic tree analysis

Hamming distances between samples were calculated using the presence and absence of somatic variants across samples. For each patient, phylogenetic tree analysis was conducted using the neighbor-joining algorithm in the APE package [23].

### Tumor mutational burden (TMB) analysis

TMB was defined as the total number of somatic nonsynonymous missense mutations per megabase (Mb) [24, 25]. Germline variants were subtracted with matched normal samples [26, 27].

### Intra-tumor heterogeneity (ITH) analysis

The percentage of mutations that were branch and private mutations, which were not present in all sections of a tumor, was determined to evaluate the degree of ITH [28, 29].

### Jaccard similarity coefficient

The Jaccard similarity coefficient was defined as the proportion of intersections between samples A and B divided by the proportion of their union [30–33]:

$${{{{{{{\mathrm{Jac}}}}}}}}\left( {{{{{{{{\mathrm{A,B}}}}}}}}} \right) = \frac{{\left| {A \cap B} \right|}}{{\left| {A \cup B}\right|}}$$

### Mutational signature analysis

Mutational signatures were determined using Mutalisk in which mutations were decomposed by linear regression [34]. The somatic variants filtered with paired normal variants were pooled according to lung cancer signatures 1, 2, 4, 5, 6, 13, 15, and 17 in the Catalogue of Somatic Mutations in Cancer database. The mutational signatures were validated by signeR based on a non-negative matrix factorization algorithm [35, 36]. The mutational signature spectrum was compared and confirmed considering cosine similarity with same as above lung signatures.

### Purity and copy number variation (CNV) analysis

Somatic CNVs were identified using a CNVkit with the default settings [37]. The circular binary segmentation method was used to integrate noisy copy number signals into the same copy number. Mutations’ normalized copy ratios and b allele frequencies were used to estimate tumor purity and ploidy using absCNseq [38]. This purity information was then used to identify the final CNVs for each sample using the CNVkit’s purity analysis function.

## Results

### Clinical characteristics

Out of 1,948 chemotherapy-naïve patients who underwent surgical resection for invasive adenocarcinoma of the lung, 132 (6.8%) were diagnosed with IMA. Eight patients with synchronous multifocal IMAs resected by surgery were enrolled in this study and designated as P01–P08, respectively. Patient characteristics are summarized in Table 1. The median age was 59 years and the ages ranged from 36 to 79 years. Two were male and six were female. Three patients were ex-smokers and five had never smoked. P01 and P06 had undergone lung transplantation due to clinically diffuse interstitial pneumonia. Subsequent pathologic examination of the explanted lungs revealed IMAs involving multiple lobes in the background of usual interstitial pneumonia and fibrosing non-specific interstitial pneumonia, respectively. In P07, pathological examination of non-neoplastic lung showed findings consistent with usual interstitial pneumonia. In the remaining patients, no evidence of fibrosing interstitial pneumonia was observed. P02 was excluded during sequencing due to the poor quality of extracted DNA.

**Table 1:** Patient cohort.

Patient No.	Age	Sex	Smoking history	Main tumor size (cm)	Stage	Location
P01	53	M	Ex-smoker	>10	IV	RUL, RML, RLL, LUL, LLL
P02	72	F	Never-smoker	8.6	IV	RUL1, RUL2, RUL3, LUL
P03	79	F	Never-smoker	6.7	IIIB	RLL1, RLL2, RLL3
P04	65	M	Ex-smoker	3.5, 1.0	IIIA	RLL, RUL1, RUL2
P05	65	F	Never-smoker	5.8	IIB	RLL1, RLL2, RLL3
P06	36	F	Ex-smoker	>10	IV	RUL, RML, LUL, LN
P07	52	F	Never-smoker	5.6, 3.2	IIB	LLL, RLL
P08	48	F	Never-smoker	5.5	IIB	LLL1, LLL2, LLL3, LLL4

*RUL* right upper lobe, *RML* right middle lobe, *RLL* right lower lobe, *LUL* left upper lobe, *LLL* left lower lobe, *LN* lymph node metastasis.

### Genomic landscape of IMAs

**Fig. 1:**
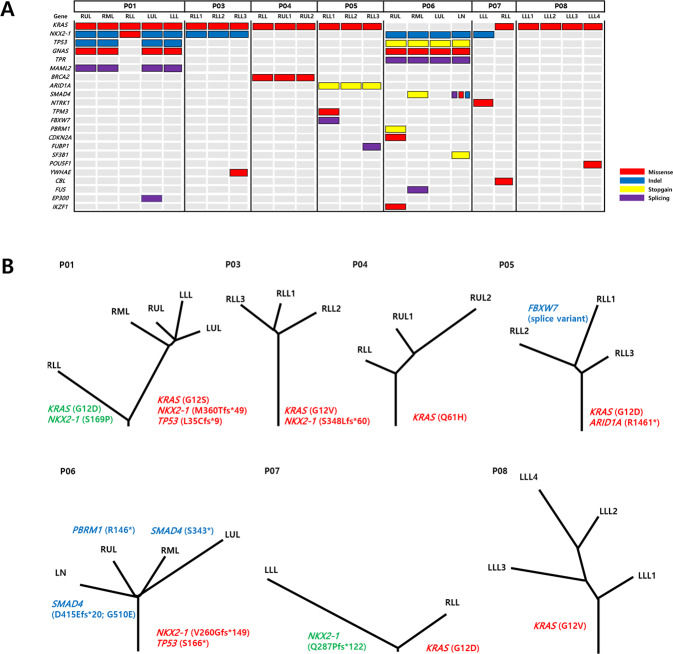
Landscape of clonal and subclonal cancer-related mutations in IMAs. **A** OncoPrint heatmap of mutations in IMAs. **B** Phylogenetic trees generated for seven patients with multifocal IMAs (letters in red: trunk mutations, letters in green: mutation in contralateral lower lobes, letters in blue: branch mutations). (RUL right upper lobe, RML right middle lobe, RLL right lower lobe, LUL left upper lobe, LLL left lower lobe, LN lymph node metastasis, Indel insertion and deletion mutation).

In total, 2–5 regions per patient were sequenced for a total of 24 sectors (Fig. 1). Sequencing occurred at a mean depth of 207×. A total of 4,059 somatic single nucleotide variants and small insertion or deletion mutations were identified with an average of 169.2 mutations/region. Mutation heat maps and phylogenetic trees were generated (Fig. 1).

All *KRAS* mutations, namely G12D, G12S, G12V, and Q61H, were confirmed to be truncal events as they were present in all sectors of the tumors, underscoring *KRAS* mutations’ role as an early driver of tumor initiation (Fig. 1 and Supplementary Table S1). However, *KRAS* mutations were different between synchronous tumors in the bilateral lower lobes of individuals (G12S vs. G12D in P01 and wild type vs. G12D in P07) (Fig. 1 and Supplementary Table S1). The second most frequently mutated gene was *NKX2-1*, found in four out of seven tumors (Fig. 1 and Supplementary Table S1) and was also a truncal event. *NKX2-1* mutations differed between bilateral lower lobes in individuals (e.g., M360Tfs*49 vs. S169P in P01 and Q287Pfs*122 vs. wild type in P07) (Fig. 1). *TP53* mutations in P01 and P06 were also truncal mutations (Fig. 1). In addition, *ARID1A* R1461* mutation was a truncal mutation in P05 (Fig. 1). The mutational status with variant allele frequencies for each patient is described in Supplementary Table S1.

### TMB of IMAs

The mean TMB was 1.13/Mb and the TMB range was 0.33–1.63 Mb (Table 2). The TMB values of IMAs were considerably lower than those of non-small-cell lung cancer, which have been reported to be 3–8/Mb [39, 40].

**Table 2:** Tumor mutational burden (TMB) of multifocal IMAs.

Patient	Tumor location	TMB
P01	RUL	1.13
	RML	0.92
	RLL	0.74
	LUL	1.04
	LLL	1.28
P03	RLL1	0.86
	RLL2	0.89
	RLL3	1.01
P04	RLL	0.77
	RUL1	1.10
	RUL2	1.48
P05	RLL1	1.39
	RLL2	1.63
	RLL3	1.34
P06	RUL	1.04
	RML	0.89
	LUL	1.13
	LN	1.25
P07	LLL	0.74
	RLL	0.33
P08	LLL1	0.56
	LLL2	0.86
	LLL3	0.80
	LLL4	0.98

*RUL* right upper lobe, *RML* right middle lobe, *RLL* right lower lobe, *LUL* left upper lobe, *LLL* left lower lobe, *LN* lymph node metastasis.

### ITH index and Jaccard coefficient of IMAs

**Fig. 2:**
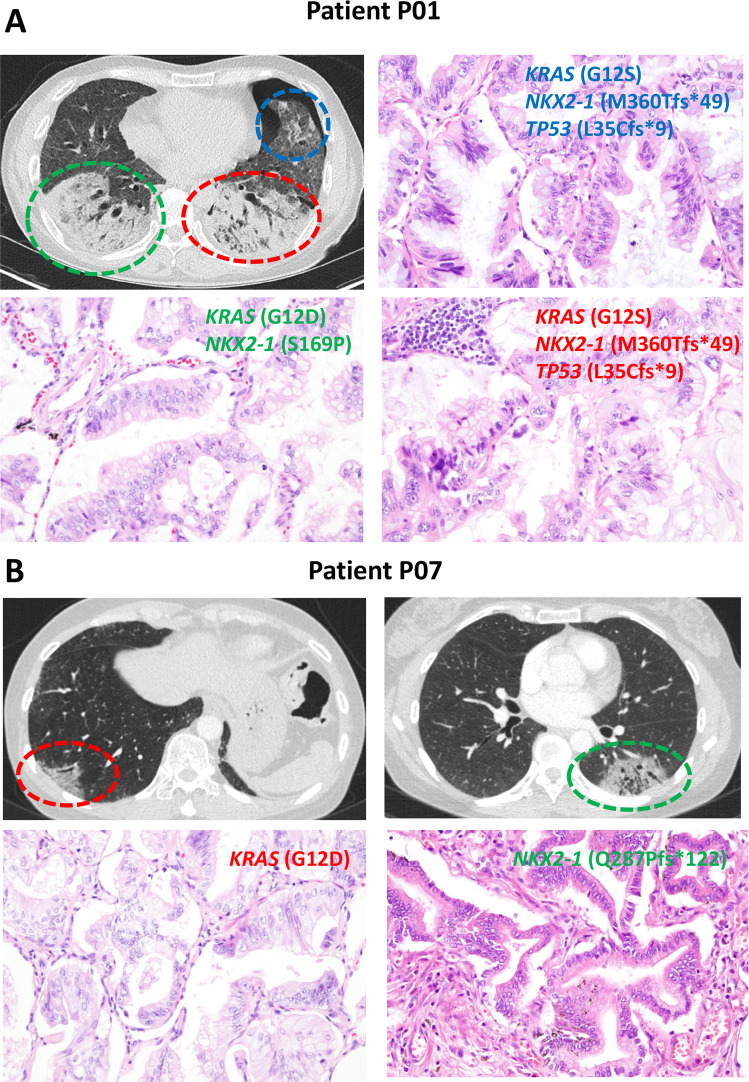
Representative radiologic and histologic findings of IMAs in P01 and P07. **A** The radiologic features of patient P01 show lobar consolidations with ground-glass opacities in bilateral lobes (right lower lobe-green color; left lower lobe-red color; left lingular segment-blue color). The tumors in the left lower lobe and left upper lobe share the same truncal mutations (*KRAS* G12S, *NKX2-1* M360Tfs*49, and *TP53* L35Cfs*9). The right lower lobe tumor is clonally distinct, harboring *KRAS* G12D and *NKX2-1* S169P mutations. **B** In P07, a mass-like ground-glass opacity with traction bronchiolectasis and internal low attenuation is observed in the subpleural area of the left lower lobe (green color), and diffuse subpleural ground-glass opacity is observed in the subpleural areas of the right lower lobe (red color). These tumors are clonally unrelated. Original magnification of histologic sections, 20× objective.

The tumors had a median ITH index of 68.1%, which was significantly higher than the previously reported 30% ITH index in lung adenocarcinoma (Table 3) [28, 29]. The median Jaccard similarity coefficient ranged from 0.16 to 0.58 except for P07 (Table 4). In P07, the Jaccard similarity coefficient was 0.01. P07’s synchronous tumors in the bilateral lower lobes had different truncal mutations (Fig. 1). The Jaccard similarity coefficient for the synchronous bilateral lower lobe tumors in P01 was 0.01, which also differed in terms of truncal events (Figs. 1 and 2).

**Table 3:** Percentage of truncal and branch mutations in IMA patients.

	Branch mutations	Truncal mutations	Percentage of branch mutations (%) (branch mutations/total mutations)
P01	37	37	50
P03	30	31	49.1
P04	112	24	82.3
P05	68	36	65.4
P06	78	25	75.7
P07	42	0	N/A
P08	41	17	70.7

**Table 4:** Jaccard similarity coefficients based on exome sequencing.

Patient	Tumor location						Median
P01		RUL	RML	RLL	LUL	LLL	
	RUL	1.00					0.50
	RML	0.52	1.00				
	RLL	0.01	0.01	1.00			
	LUL	0.60	0.55	0.01	1.00		
	LLL	0.53	0.49	0.01	0.58	1.00	
P03		RLL1	RLL2	RLL3			
	RLL1	1.00					0.52
	RLL2	0.52	1.00				
	RLL3	0.59	0.48	1.00			
P04		RLL	RUL1	RUL2			
	RLL	1.00					0.26
	RUL1	0.26	1.00				
	RUL2	0.21	0.38	1.00			
P05		RLL1	RLL2	RLL3			
	RLL1	1.00					0.29
	RLL2	0.29	1.00				
	RLL3	0.30	0.27	1.00			
P06		RUL	RML	LUL	LN		
	RUL	1.00					0.30
	RML	0.35	1.00				
	LUL	0.27	0.26	1.00			
	LN	0.34	0.32	0.23	1.00		
P07		LLL	RLL				
	LLL	1.00					0.01
	RLL	0.01	1.00				
P08		LLL1	LLL2	LLL3	LLL4		
	LLL1	1.00					0.24
	LLL2	0.20	1.00				
	LLL3	0.19	0.31	1.00			
	LLL4	0.16	0.47	0.28	1.00		

*RUL* right upper lobe, *RML* right middle lobe, *RLL* right lower lobe, *LUL* left upper lobe, *LLL* left lower lobe, *LN* lymph node metastasis.

### Mutational signatures of IMAs

**Fig. 3:**
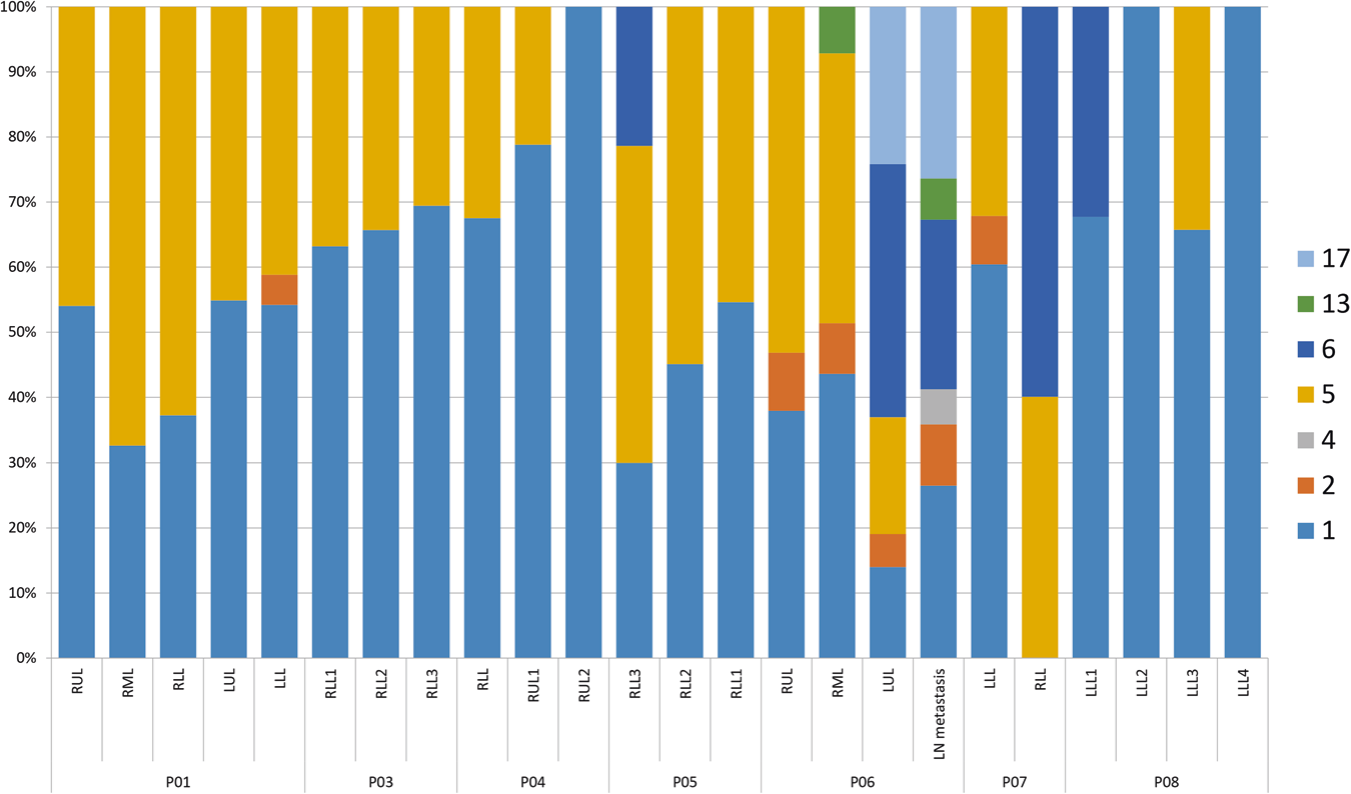
Mutational signatures of multifocal IMAs. The right axis of the graph is arranged in accordance with the heatmap in Fig. 1. Signature 1 is the result of an endogenous mutational process. Signature 2 has been attributed to the activity of the AID/APOBEC family of cytidine deaminases. Signature 4 is associated with smoking. The etiology of signature 5 is unknown. Signature 6 is associated with defective DNA mismatch repair. Signature 13 has been attributed to the activity of the AID/APOBEC family of cytidine deaminases converting cytosine to uracil. The etiology of signature 17 remains unknown. Signature 1 is found in almost all samples (95.8%, 23/24). Signatures 2 and 13 were found in six samples (25.0%). Signature 6 was found in five out of 24 samples (20.8%).

Among seven mutation signatures identified in our samples (Fig. 3), signature 1, the result of an endogenous mutational process, was found in almost all samples (95.8%, 23/24). Signatures 2 and 13, APOBEC-related signatures, were found in six samples (25.0%). Signature 6, associated with defective DNA mismatch repair (MMR), was found in five out of 24 samples (20.8%). Signature 4, a smoking-related signature, was observed in only one sample with a small portion.

### CNVs of IMAs

Genome-wide CNVs showed distinct patterns between patients, and no recurrent CNVs were present in all sectors of the tumors (Supplementary Fig. S1, S2). CNVs observed in each patient provided the evidence of the clonal relationship in some cases. In P01, some CNVs (such as chromosome 12p duplication, and deletion of chromosome 17p and 19p) were identified in tumors of the left lower lobe, left upper lobe, and right upper lobe (Supplementary Fig. S2A). CNVs in chromosomes 9 and 14 were also present in all sectors of P05 (Supplementary Fig. S2B). In P06, loss of chromosome 9 and partial duplication of chromosomes 6, 8, and 17 were present in all tumor sectors (Supplementary Fig. S2C). We also found that amplifications of *KRAS* and *ERBB2* gene were present only in the lymph node metastasis and right middle lobe of P06, respectively.

## Discussion

We have characterized the genomic architecture of multifocal IMAs through multi-region whole exome sequencing. *KRAS*, *NKX2-1*, *TP53, or ARID1A* mutations were found in all multifocal IMAs, serving as genetic evidence for the occurrence of intrapulmonary metastases. *KRAS* mutations account for 60–70% of IMA driver mutations [5, 9, 41]. In this study’s cohort, various *KRAS* mutations (G12D, G12V, G12S, and Q61H) were identified, of which three were G12D and two were G12V. This number of instances was small, but their frequency was consistent with that found in previous studies [5, 9]. Recent studies on *KRAS* mutations have reported encouraging results [42]. IMA patients with *KRAS* G12D mutation responded exceptionally well to the proteasome inhibitor bortezomib [43]. Thus, it may present new therapeutic opportunities for patients with IMA.

Previous studies have reported *NKX2-1* mutations in IMAs [6]. *NKX2-1*, also known as *TTF- 1*, plays role in lung morphogenesis and differentiation [44]. *NKX2-1* is an organ-specific marker and is expressed in most lung primary adenocarcinomas, so its immunohistochemistry is commonly used to differentiate between primary lung tumors and metastatic neoplasm [45]. Recurrent *NKX2-1* mutations have been observed in IMAs, which suggests that *NKX2-1* plays a role in IMA carcinogenesis [6]. However, this study was the first to observe the truncal nature of *NKX2-1* mutations. This study’s results suggest that *NKX2-1* mutations may be involved in the early carcinogenesis of IMA. Of the five types of *NKX2-1* mutations found in this study, four were frameshift mutations, which was consistent with the results of previous studies [6, 46]. Transgenic mice with inactivated Nkx2-1/Ttf-1 develop mucinous adenocarcinomas in the lungs that resemble human IMAs [47]. Taken together, these findings suggest that *NKX2-1* mutations play a pivotal role in the development of IMA.

A nonsense mutation R1461* in *ARID1A* gene was found in all separate areas of P05, implying a truncal mutation. As Hung and colleagues reported, we thought that loss of function mutations of *ARID1A* is also clonal [48].

In this study, two patients presented with tumor involvement in their bilateral lower lobes. Unexpectedly, the genetic types of *KRAS* and *NKX2-1* mutations differed in each lobe, suggesting that these tumors were clonally unrelated. Since no *KRAS* mutation was found in the left lower lobe tumor of patient P07, we went through the following confirmation process. First, we manually reviewed sequencing reads for the *KRAS* mutation, but no *KRAS* mutation was found. Second, we also performed pyrosequencing (Qiagen, Hilden, Germany) and PNA clamp-based real-time PCR (Panagene, Daejeon, Korea), but no *KRAS* mutation was found. Third, we further analyzed the mitochondrial mutations, and the results supported that the tumors in the lower lobes of P01 and P07 were clonally unrelated to each other (Supplementary Fig. S3). We have noticed that the two patients had background usual interstitial pneumonia. It is known that underlying usual interstitial pneumonia may predispose to the formation of multiple IMAs [49, 50]. This result indicates that synchronous multifocal IMAs can rarely occur in multicentric origins, especially in the background of fibrosing interstitial pneumonia. We recently experienced the same type of *KRAS* mutation (G12V) in a patient with metachronous multifocal IMAs involving the left lower lobe and then the right lower lobe (data not shown). Taking these findings together, caution is required when staging these tumors, and in some cases, mutational analysis can be helpful. We previously found that approximately 75% of IMA patients had primary masses in their lower lobes [41]. This result may indicate that IMAs, which spread to multiple lobes, including the lower lobes, are likely to originate from the lower lobe in probability. In this cohort, six out of seven patients (86%) had IMAs involving the lower lobe.

*TP53* mutations were found in the IMAs of two patients who presented with the involvement of multiple lobes and were given poor prognoses. There is accumulating evidence that co-occurring mutations can affect the biological behavior of lung cancers [51]. Recent studies have found that treatment, patient prognosis, and responses to targeted therapy or immunotherapy may differ according to these subtypes [52, 53]. Although the number of cases is limited, the presence of *TP53* mutations in IMA suggests a more aggressive biological behavior, as demonstrated in other lung cancers [54].

In this study, IMAs had a low somatic mutation burden, but they did have a heterogeneous genomic landscape, which was similar to Asian *EGFR*-mutant lung adenocarcinomas [54]. In contrast, Caucasian lung cancers have a high-mutation burden and low intra-tumor heterogeneity [28, 29]. The median proportion of branch mutation in smoker-dominated Caucasian lung adenocarcinomas was about 30%. In contrast, the proportion of branch mutation in Asian *EGFR-*mutant adenocarcinomas was 62.3%, which was similar to that of IMAs in our cohort. In addition, from TMB analysis, it appears even clonally related tumors can differ somewhat in TMB counts. Both intratumor genetic heterogeneity and tumor purity can affect the TMB counts [55]. In this study, since whole exome sequencing was performed on samples having a tumor purity of 20% or more, we thought that the main factor affecting TMB counts was intratumor genetic heterogeneity. However, it cannot be excluded that the low tumor purity may have affected the TMB counts in some cases.

The mutational signature analysis found that IMAs were predominantly associated with endogenous mutational process (signature 1), APOBEC activity (signatures 2 and 13), and MMR deficiency (signature 6), not related to smoking. These findings suggest that IMAs are distinguished from conventional adenocarcinomas or *KRAS*-mutant non-mucinous adenocarcinomas, which are closely related to smoking signatures [56]. In addition, the prevalence of APOBEC-related signatures can contribute to subclonal diversification and ITH of IMAs found in this study [30]. Interestingly, MMR deficiency signature was found in five of 24 areas. We performed immunohistochemistry for MMR proteins (MLH1, MSH2, MSH6, and PMS2) and microsatellite instability (MSI) RT-PCR test using U-TOP MSI Detection Kit (SEASUN Biomaterials, Daejeon, Korea) for those samples. No MSI-high tumors were found, and all MMR proteins were preserved. According to reports so far, the frequency of MSI-high in lung cancer is very low, ranging from 0.5 to 0.8% [57, 58]. On the other hand, the MMR deficiency signature has been reported in in situ or early lung adenocarcinoma, and lung adenocarcinomas with MMR deficiency signature were reported to have a better prognosis compared to tumors with APOBEC signature [59]. We think that it is necessary to distinguish between a tumor with MMR deficiency signature and an MSI-high tumor. Because these cases are not an MSI-high tumor, it is not considered to be a candidate for cancer immunotherapy. Further studies are necessary to elucidate the clinical and biological significance of MMR deficiency signature in lung cancers.

This study has several limitations. Due to the limited sample size, the clinical impact or significance of genomic findings in multifocal IMAs has not been investigated. Also, since low tumor purity is a well-known issue with the sequencing of IMAs, the possibility that low tumor purity may have affected the genomic results in some cases cannot be completely excluded.

In conclusion, genomic evidence found in this study indicated the clonal intrapulmonary spread of diffuse pneumonic-type or multifocal IMAs, although they can occur in multicentric origins in the background of usual interstitial pneumonia. IMAs exhibited a heterogeneous genomic landscape despite the low somatic mutation burden. Further studies are warranted to determine the clinical significance of the genomic characteristics of IMAs in expanded cohorts.

## Supplementary information

Supplementary data

## Data Availability

All data generated or analyzed during this study are included in this published article and its supplementary information files.
