# Supplementary material for: Genomic characteristics of invasive mucinous adenocarcinoma of the lung with multiple pulmonary sites of involvement
Source: Mod Pathol. 2021 Jul 21;35(2):202–9. doi: 10.1038/s41379-021-00872-0 (PMC8786658; doi:10.1038/s41379-021-00872-0)

Supplementary Data

Supplementary Table S1. Cancer-related genomic variants found in this study.

Patient No.	Tumor location *	Chromosome	Position	Gene	Variant type	HGVSc	Protein change	VAF
P01	RUL	chr12	25398285	*KRAS*	missense_variant	NM_033360.3:c.34G>A	p.Gly12Ser	0.279
	RUL	chr14	36986517	*NKX2-1*	frameshift_variant (Indel)	NM_003317.3:c.1079_1080delinsCCA	p.Met360Thrfs*49	0.187
	RUL	chr17	7579584	*TP53*	frameshift_variant	NM_000546.5:c.102delC	p.Leu35Cysfs*9	0.092
	RUL	chr20	57429949	*GNAS*	missense_variant	NM_001309883.1:c.1442G>A	p.Arg481His	0.129
	RUL	chr11	95718809	*MAML2*	splice_region_variant &intron_variant	NM_032427.3:c.2344-3A>G		0.087
	RML	chr12	25398285	*KRAS*	missense_variant	NM_033360.3:c.34G>A	p.Gly12Ser	0.286
	RML	chr14	36986517	*NKX2-1*	frameshift_variant (Indel)	NM_003317.3:c.1079_1080delinsCCA	p.Met360Thrfs*49	0.177
	RML	chr17	7579584	*TP53*	frameshift_variant	NM_000546.5:c.102delC	p.Leu35Cysfs*9	0.137
	RML	chr20	57429949	*GNAS*	missense_variant	NM_001309883.1:c.1442G>A	p.Arg481His	0.137
	RML	chr11	95718809	*MAML2*	splice_region_variant &intron_variant	NM_032427.3:c.2344-3A>G		0.069
	RLL	chr12	25398284	*KRAS*	missense_variant	NM_033360.3:c.35G>A	p.Gly12Asp	0.107
	RLL	chr14	36987094	*NKX2-1*	missense_variant	NM_003317.3:c.505T>C	p.Ser169Pro	0.058
	LUL	chr12	25398285	*KRAS*	missense_variant	NM_033360.3:c.34G>A	p.Gly12Ser	0.625
	LUL	chr14	36986517	*NKX2-1*	frameshift_variant (Indel)	NM_003317.3:c.1079_1080delinsCCA	p.Met360Thrfs*49	0.430
	LUL	chr17	7579584	*TP53*	frameshift_variant	NM_000546.5:c.102delC	p.Leu35Cysfs*9	0.279

	LUL	chr20	57429949	*GNAS*	missense_variant	NM_001309883.1:c.1442G>A	p.Arg481His	0.269
	LUL	chr11	95718809	*MAML2*	splice_region_variant &intron_variant	NM_032427.3:c.2344-3A>G		0.066
	LUL	chr22	41545024	*EP300*	splice_region_variant &intron_variant	NM_001429.3:c.2242-5_2242-4delTT		0.147
	LLL	chr12	25398285	*KRAS*	missense_variant	NM_033360.3:c.34G>A	p.Gly12Ser	0.619
	LLL	chr14	36986517	*NKX2-1*	frameshift_variant (Indel)	NM_003317.3:c.1079_1080delinsCCA	p.Met360Thrfs*49	0.463
	LLL	chr17	7579584	*TP53*	frameshift_variant	NM_000546.5:c.102delC	p.Leu35Cysfs*9	0.337
	LLL	chr20	57429949	*GNAS*	missense_variant	NM_001309883.1:c.1442G>A	p.Arg481His	0.274
	LLL	chr11	95718809	*MAML2*	splice_region_variant&intron_variant	NM_032427.3:c.2344-3A>G		0.098
P03	RLL1	chr12	25398284	*KRAS*	missense_variant	NM_033360.3:c.35G>T	p.Gly12Val	0.333
	RLL1	chr14	36986553	*NKX2-1*	frameshift_variant (Indel)	NM_003317.3:c.1043_1045delinsT	p.Ser348Leufs*60	0.158
	RLL2	chr12	25398284	*KRAS*	missense_variant	NM_033360.3:c.35G>T	p.Gly12Val	0.310
	RLL2	chr14	36986553	*NKX2-1*	frameshift_variant (Indel)	NM_003317.3:c.1043_1045delinsT	p.Ser348Leufs*60	0.108
	RLL3	chr12	25398284	*KRAS*	missense_variant	NM_033360.3:c.35G>T	p.Gly12Val	0.233
	RLL3	chr14	36986553	*NKX2-1*	frameshift_variant (Indel)	NM_003317.3:c.1043_1045delinsT	p.Ser348Leufs*60	0.140
	RLL3	chr17	1257597	*YWHAE*	missense_variant	NM_006761.4:c.623C>T	p.Thr208Met	0.047
P04	RLL	chr12	25380275	*KRAS*	missense_variant	NM_033360.3:c.183A>T	p.Gln61His	0.030
	RLL	chr13	32915219	*BRCA2*	missense_variant	NM_000059.3:c.6727T>C	p.Ser2243Pro	0.043
	RLL	chr20	43047123	*HNF4A*	missense_variant	NM_000457.4:c.707C>A	p.Ser236Tyr	0.033
	RUL1	chr12	25380275	*KRAS*	missense_variant	NM_033360.3:c.183A>T	p.Gln61His	0.022
	RUL1	chr13	32915219	*BRCA2*	missense_variant	NM_000059.3:c.6727T>C	p.Ser2243Pro	0.059
	RUL1	chr20	43047123	*HNF4A*	missense_variant	NM_000457.4:c.707C>A	p.Ser236Tyr	0.050

	RUL2	chr12	25380275	*KRAS*	missense_variant	NM_033360.3:c.183A>T	p.Gln61His	0.160
	RUL2	chr13	32915219	*BRCA2*	missense_variant	NM_000059.3:c.6727T>C	p.Ser2243Pro	0.133
	RUL2	chr20	43047123	*HNF4A*	missense_variant	NM_000457.4:c.707C>A	p.Ser236Tyr	0.106
P05	RLL1	chr12	25398284	*KRAS*	missense_variant	NM_033360.3:c.35G>A	p.Gly12Asp	0.313
	RLL1	chr1	27101099	*ARID1A*	stop_gained	NM_006015.4:c.4381C>T	p.Arg1461*	0.047
	RLL1	chr4	153268081	*FBXW7*	splice_donor_variant&intron_variant	NM_033632.3:726+1G>A		0.049
	RLL1	chr1	154131495	*TPM3*	missense_variant	NM_001043352.1:c.694C>T	p.Arg232Cys	0.032
	RLL2	chr12	25398284	*KRAS*	missense_variant	NM_033360.3:c.35G>A	p.Gly12Asp	0.273
	RLL2	chr1	27101099	*ARID1A*	stop_gained	NM_006015.4:c.4381C>T	p.Arg1461*	0.030
	RLL3	chr12	25398284	*KRAS*	missense_variant	NM_033360.3:c.35G>A	p.Gly12Asp	0.121
	RLL3	chr1	27101099	*ARID1A*	stop_gained	NM_006015.4:c.4381C>T	p.Arg1461*	0.056
	RLL3	chr1	78435701	*FUBP1*	splice_region_variant&intron_variant	NM_001303433.1:c.121-4_121-3delTT		0.107
P06	RUL	chr14	36986820	*NKX2-1*	frameshift_variant (Indel)	NM_003317.3:c.778dupG	p.Val260Glyfs*149	0.156
	RUL	chr17	7578433	*TP53*	stop_gained	NM_000546.5:c.497C>G	p.Ser166*	0.203
	RUL	chr20	57430122	*GNAS*	missense_variant	NM_080425.3:c.1802G>A	p.Arg601Gln	0.152
	RUL	chr1	186321247	*TPR*	splice_region_variant&intron_variant	NM_003292.2:c.2335-5T>A		0.185
	RUL	chr9	21974541	*CDKN2A*	missense_variant	NM_058197.4:c.286G>A	p.Val96Ile	0.030
	RUL	chr3	52696241	*PBRM1*	stop_gained	NM_018313.4:c.436C>T	p.Arg146*	0.184
	RUL	chr7	50435797	*IKZF1*	missense_variant	NM_001291845.1:c.254C>T	p.Ala85Val	0.031
	RML	chr14	36986820	*NKX2-1*	frameshift_variant (Indel)	NM_003317.3:c.778dupG	p.Val260Glyfs*149	0.286

	RML	chr17	7578433	*TP53*	stop_gained	NM_000546.5:c.497C>G	p.Ser166*	0.278
	RML	chr20	57430122	*GNAS*	missense_variant	NM_080425.3:c.1802G>A	p.Arg601Gln	0.257
	RML	chr1	186321247	*TPR*	splice_region_variant&intron_variant	NM_003292.2:c.2335-5T>A		0.214
	RML	chr18	48591865	*SMAD4*	stop_gained	NM_005359.5:c.1028C>G	p.Ser343*	0.035
	RML	chr16	31202698	*FUS*	splice_region_variant&intron_variant	NM_004960.3c.1542-6_1542-5delTT		0.182
	LUL	chr14	36986820	*NKX2-1*	frameshift_variant (Indel)	NM_003317.3:c.778dupG	p.Val260Glyfs*149	0.120
	LUL	chr17	7578433	*TP53*	stop_gained	NM_000546.5:c.497C>G	p.Ser166*	0.240
	LUL	chr20	57430122	*GNAS*	missense_variant	NM_080425.3:c.1802G>A	p.Arg601Gln	0.167
	LUL	chr1	186321247	*TPR*	splice_region_variant&intron_variant	NM_003292.2:c.2335-5T>A		0.294
	LN	chr14	36986820	*NKX2-1*	frameshift_variant (Indel)	NM_003317.3:c.778dupG	p.Val260Glyfs*149	0.210
	LN	chr17	7578433	*TP53*	stop_gained	NM_000546.5:c.497C>G	p.Ser166*	0.269
	LN	chr20	57430122	*GNAS*	missense_variant	NM_080425.3:c.1802G>A	p.Arg601Gln	0.220
	LN	chr1	186321247	*TPR*	splice_region_variant&intron_variant	NM_003292.2:c.2335-5T>A		0.200
	LN	chr18	48575054	*SMAD4*	splice_acceptor_variant&intron_variant	NM_005359.5:c.250-2A>T	-	0.091
	LN	chr18	48593490	*SMAD4*	frameshift_variant	NM_005359.5:c.1245_1248delCAGA	p.Asp415Glufs*20	0.093
	LN	chr18	48604707	*SMAD4*	missense_variant	NM_005359.5:c.1529G>A	p.Gly510Glu	0.044
	LN	chr2	198257896	*SF3B1*	stop_gained	NM_012433.3:c.3556C>T	p.Gln1186*	0.044
P07	LLL	chr14	36986739	*NKX2-1*	frameshift_variant (Indel)	NM_003317.3:c.859dupC	p.Gln287Profs*122	0.114
	LLL	chr1	156845395	*NTRK1*	missense_variant	NM_002529.3:c.1438G>A	p.Glu480Lys	0.085
	RLL	chr12	25398284	*KRAS*	missense_variant	NM_033360.3:c.35G>A	p.Gly12Asp	0.030

	RLL	chr11	11917030<br>6	*CBL*	missense_variant	NM_005188.3:c.2536C>T	p.Pro846Ser	0.070
P08	LLL1	chr12	25398284	*KRAS*	missense_variant	NM_033360.3:c.35G>T	p.Gly12Val	0.109
	LLL2	chr12	25398284	*KRAS*	missense_variant	NM_033360.3:c.35G>T	p.Gly12Val	0.196
	LLL3	chr12	25398284	*KRAS*	missense_variant	NM_033360.3:c.35G>T	p.Gly12Val	0.194
	LLL4	chr12	25398284	*KRAS*	missense_variant	NM_033360.3:c.35G>T	p.Gly12Val	0.229
	LLL4	chr6	31133449	*POU5F1*	missense_variant	NM_002701.5.6:c.556C>T	p.Arg186Cys	0.032

* RUL: right upper lobe, RML: right middle lobe, RLL: right lower lobe, LUL: left upper lobe, LLL: left lower lobe, LN: lymph node metastasis.

Supplementary Figure S1. Results of copy number analysis of multifocal IMAs. Light red (▬) indicates a gain in one copy over the ploidy and deep red (▬) indicates a gain in more than two copies beyond the ploidy. Light blue (▬) indicates a loss beyond the ploidy and deep blue (▬) indicates a loss of more than two copies beyond to the ploidy.

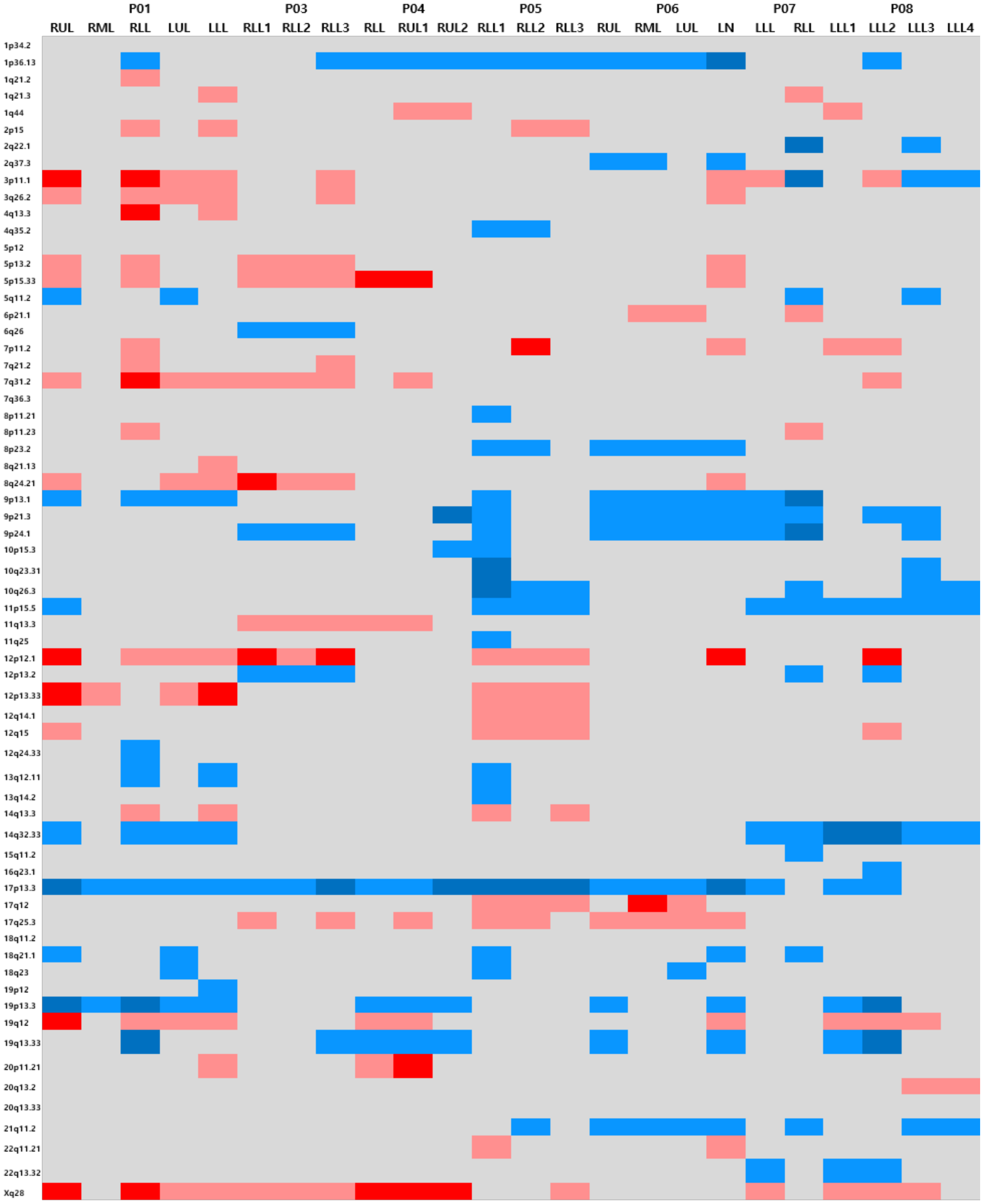

Supplementary Figure S2. Genome-wide copy number ratio and b-allele frequency of P01 (A), P05 (B), and P06 (C). Highlights with green color indicate clonally altered regions.

A

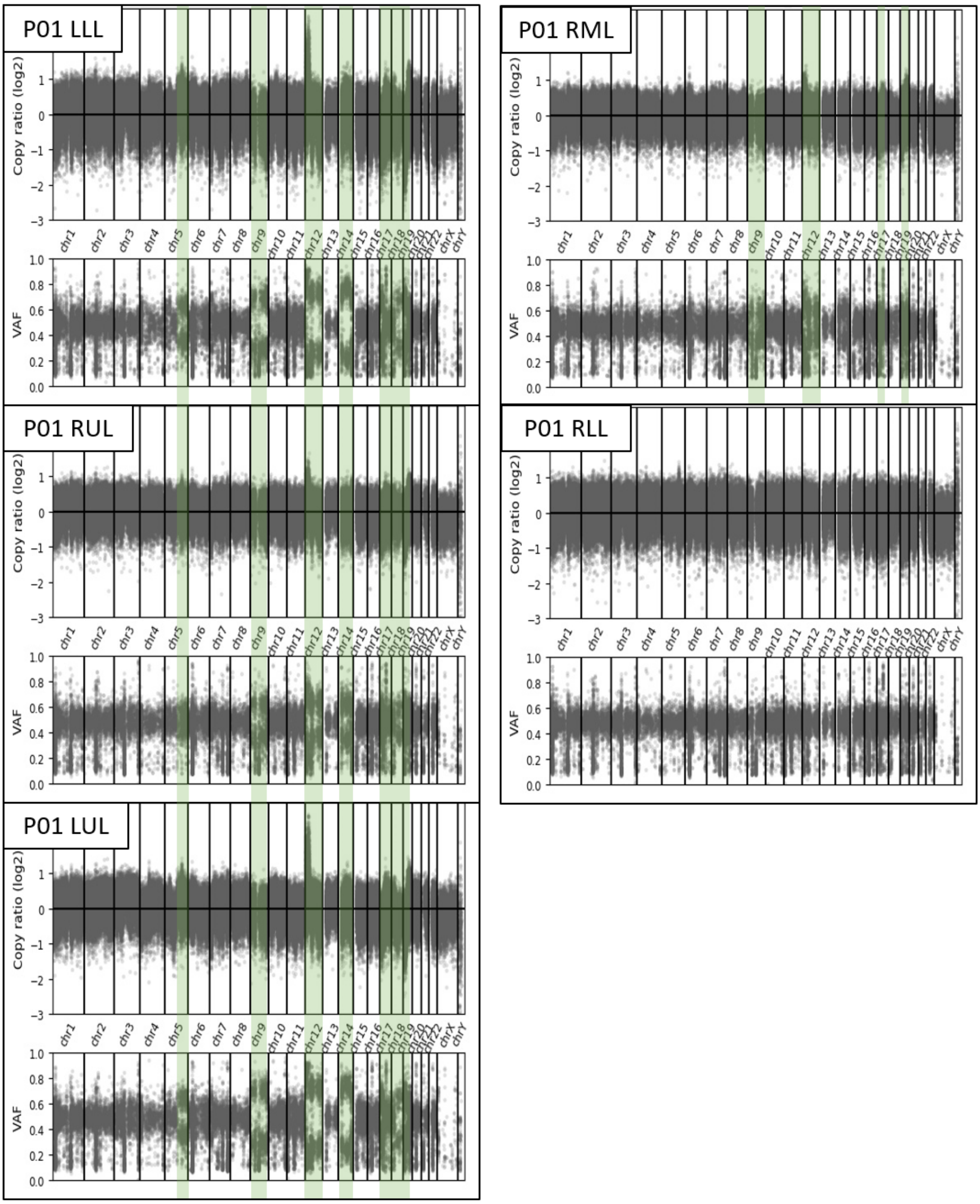

B

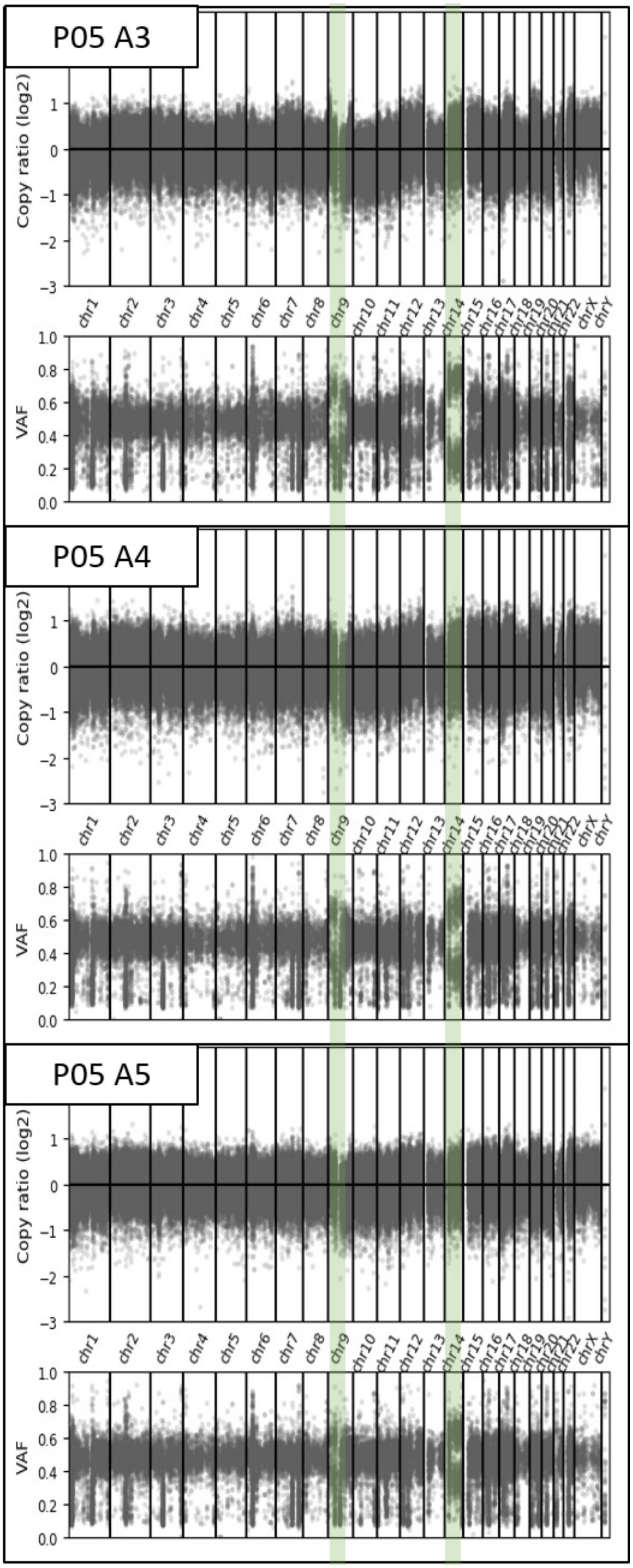
P05 A3
Copy ratio (log2)
VAF
P05 A4
Copy ratio (log2)
VAF
P05 A5
Copy ratio (log2)
VAF
chr1
chr2
chr3
chr4
chr5
chr6
chr7
chr8
chr9
chr10
chr11
chr12
chr13
chr14
chr15
chr16
chr17
chr18
chr19
chr20
chr21
chr22
chrX
chrY

C

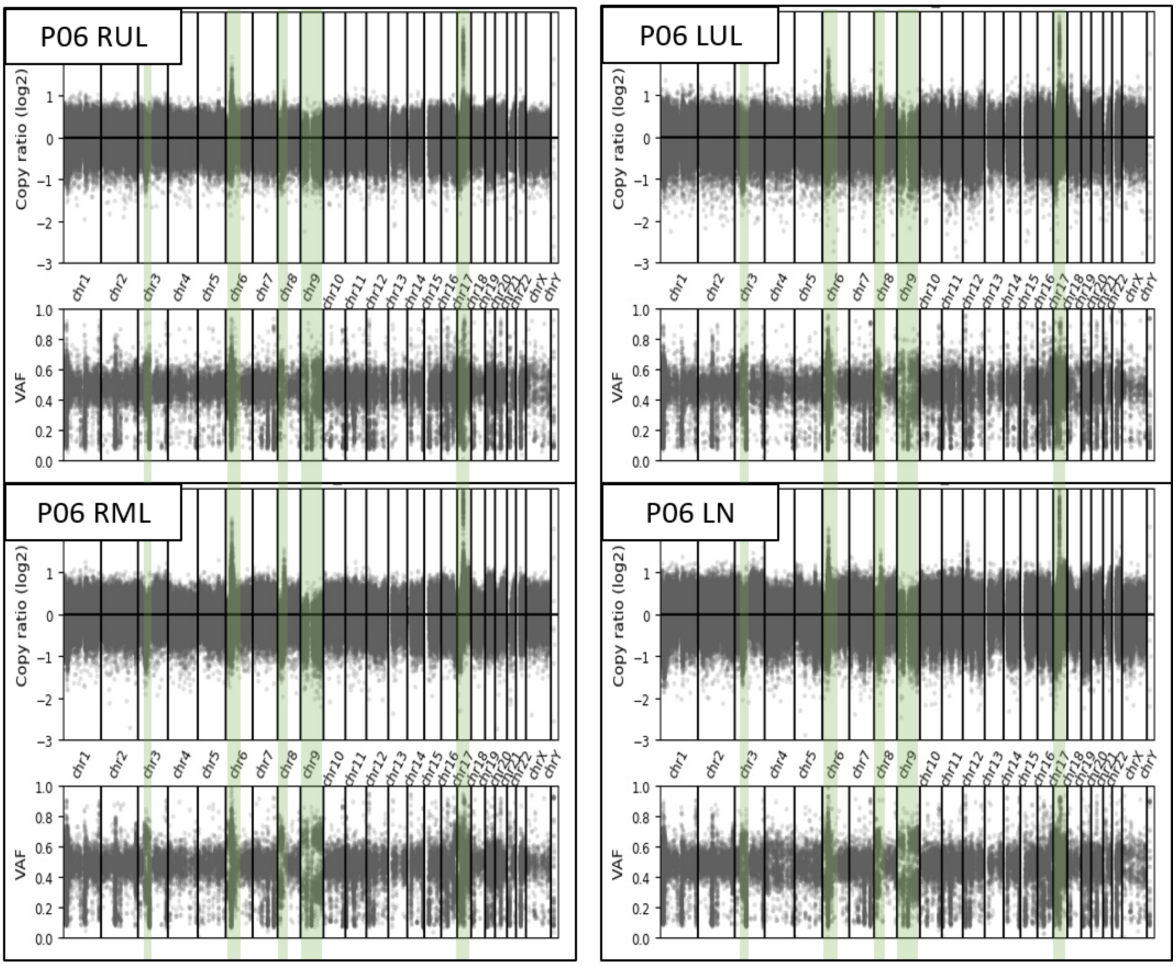

P06 RUL
P06 LUL
P06 RML
P06 LN
Copy ratio (log2)
VAF
chr1
chr2
chr3
chr4
chr5
chr6
chr7
chr8
chr9
chr10
chr11
chr12
chr13
chr14
chr15
chr16
chr17
chr18
chr19
chr20
chr21
chr22
chrX
chrY

Supplementary Figure S3. Distribution of mitochondrial mutations in P01 (A) and P07 (B). A: chrM 11867 is present in all lobes except for RLL, suggesting that the RLL tumor is clonally distinct. B: A common mitochondrial mutation is not observed between tumors of LLL and RLL.

A

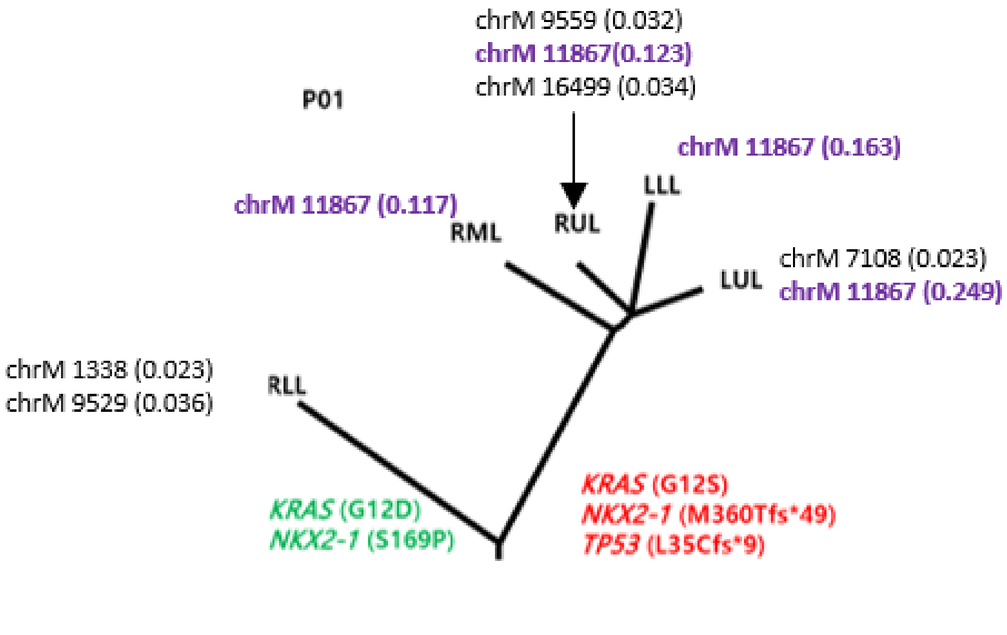

B

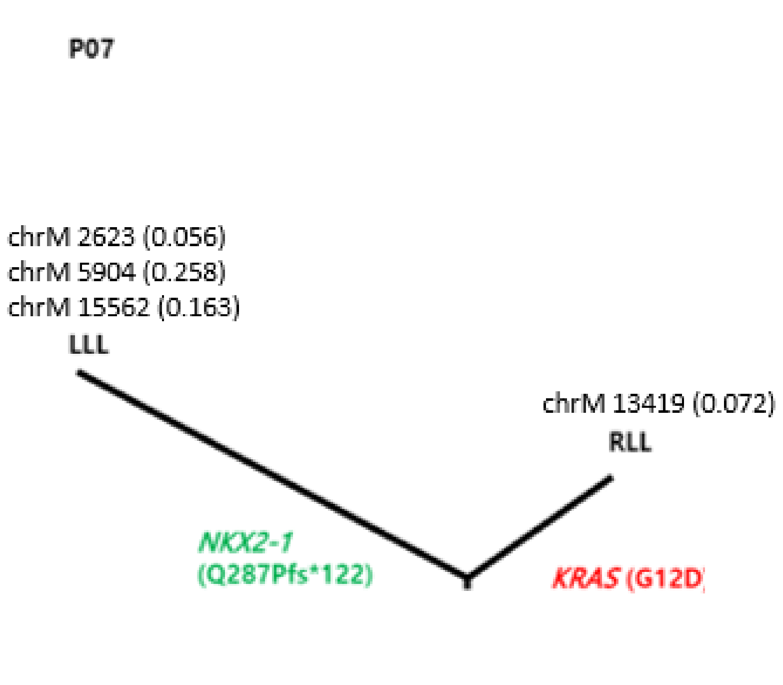

Supplement: Supplementary file 1 — Supplementary data [file 41379_2021_872_MOESM1_ESM.pdf]
